# Application of test-enhanced learning (TEL) in obstetrics and gynecology: a prospective study

**DOI:** 10.1007/s00404-022-06656-4

**Published:** 2022-07-13

**Authors:** Florian Recker, Nicolas Haverkamp, Alexander Mustea, Ulrich Gembruch, Tobias Raupach

**Affiliations:** 1 Department of Obstetrics and Gynecology, University Hospital Bonn, Venusberg Campus 1, 53127 Bonn, Germany; 2 Institute for Medical Education, University Hospital Bonn, Venusberg Campus 1, 53127 Bonn, Germany; 3 Student’s Dean Office, University Hospital Bonn, Venusberg Campus 1, 53127 Bonn, Germany; 4 Department of Gynecology and Gynecological Oncology, University Hospital Bonn, Venusberg Campus 1, 53127 Bonn, Germany; 5 Department of Obstetrics and Prenatal Medicine, University Hospital Bonn, Venusberg Campus 1, 53127 Bonn, Germany

**Keywords:** Test-enhanced learning, Gynecology, Obstetrics, Medical education

## Abstract

**Objective:**

Clinical reasoning is an essential skill, the foundations of which should be acquired during medical school. Within the format of test-based learning, such examinations can also be used to support the long-term retention of procedural knowledge necessary for clinical reasoning. The aim was to investigate whether repeated exposure to clinical cases in obstetrics and gynecology (OBGYN) with built-in questions leads to higher learning outcome than pure reading cases and what influence the delay between the intervention and the final test has on the retention of the respective content.

**Methods:**

In this non-randomised crossover study, 5th-year medical students (duration of the study is 6 years) taking a 1-week clinical attachment in OBGYN participated in computer-based case seminars in winter term 2020/2021, in which different case histories on gynecological-obstetric diseases were presented. Case content was identical for all groups, but the presentation format (cases with key feature questions vs read-only cases) of individual case vignettes changed weekly. The also intervention was repeated after 2 weeks for each group. Knowledge was assessed in an entry and an exit exam consisting of 40 short-answer questions.

**Results:**

A total of 94 out of 118 eligible students participated in the study (response rate: 79.7%). Learning outcome was significantly higher for items presented in the key feature format compared to items presented as read-only cases (74.2 ± 8.6% vs. 71.0 ± 9.2%; *p* = 0.017). Furthermore, the analysis showed that the temporal distance of the intervention package from the final examination had no influence on retention.

**Conclusion:**

This is the first study to demonstrate an effect of test-enhanced learning on clinical reasoning in the subject of OGBYN. In this cross-over study, repeated testing was more effective than repeated case-based learning alone. Curricular implementation of longitudinal key feature testing can thus improve learning outcomes for OBGYN.

## What does this study add to the clinical work

**Table:** 

This study is the first to investigate the effectiveness of test-enhanced learning in the field of gynaecology and obstetrics (OBGYN). Repeated testing was more effective than repeated case-based learning in OBGYN. Curricular implementation of longitudinal key feature testing can thus improve learning outcomes for OBGYN.	

## Introduction

Undergraduate medical education's ultimate goal is to provide future physicians with a sound knowledge basis and the necessary abilities to manage their patients. The process of applying information to new clinical settings in order to make informed decisions regarding diagnostic procedures and treatment alternatives is one of the higher order cognitive tasks that must be mastered.

In this context, clinical reasoning is an essential competence in medical education [1]. It describes the making of informed decisions by a physician based on knowledge, intuition, experience, and guidelines about the diagnostic and therapeutic procedure based on a patient’s initial situation and symptoms, taking into account potential possible differential diagnoses. Two cognitive approaches are currently described in the literature that underlie clinical reasoning, namely the intuitive and the analytical approach. Overall, a combination of both approaches is realistic and important for clinical reasoning [2].

Thus, clinical reasoning is an essential skill, the foundations of which must be acquired during basic medical training. Students’ performance in clinical reasoning can be assessed summatively by means of key feature questions [3].

In 1995, Page et al. [4] introduced the concept of a key feature and described its function as the cornerstone of key feature problems, a new format for the written assessment of medical students’ and practitioners’ clinical decision-making skills. A key feature is defined as a critical step in solving a clinical problem, and a key feature problem consists of a clinical case scenario followed by questions focusing only on these critical steps (key-feature question/KFQ). Hrynchak et al. [5] summarised the evidence on the reliability and validity of KFQs for assessing clinical reasoning and were able to show, based on a systematic literature review, that KFQs are an adequate format for assessing clinical reasoning. Taken together with the current evidence on test-enhanced learning, there is scope for using formative examinations made up of KFQs in order to enhance clinical reasoning competencies.

Test-enhanced learning represents a pedagogical intervention that is consistent with the current emphasis on using assessment to improve pedagogical practice in medical education [6]. It represents a fitting complement to the tools that educators can use to help medical students, residents and practicing physicians retain information and progress towards greater clinical expertise [7]. In doing so, this can also be implemented into digital teaching in the current Covid 19 times [8]. The available research demonstrates robust effects across health professions, learners, learning formats, and learning outcomes.

The application of test-enhanced learning in the field of gynecology and obstetrics has not yet been investigated. Thus, the following questions arise:

- Does repeated exposure to clinical cases in OBGYN with interspersed key feature questions (KF cases) lead to better learning outcome than repeated exposure to read-only cases (RO cases) with the same content but without questions?
- What influence does the temporal distance of the intervention package from the final examination have on the retention of the respective contents?

The following hypotheses are formulated for the primary study question:

- H0: There is no difference when clinical cases with built-in questions are used instead of cases with the same content but without built-in questions.
- H1: There is a difference when clinical cases with built-in questions are used instead of cases with the same content but without built-in questions.

For the secondary question, the following hypotheses are formulated:

- H0: The temporal distance of the intervention package from the final exam has no effect on the overall outcome and effectiveness of exam-supported or unsupported learning.
- H1: The temporal distance of the intervention package from the final examination has an effect on the overall outcome and the effectiveness of examination-supported or unsupported learning.

## Methods

This was a non-randomised, controlled, non-blinded crossover trial involving medical students in the fifth year of medical school. Undergraduate medical education is separated into two phases at the institution where the study was done. Basic sciences, as well as anatomy, biochemistry, physiology, and medical psychology, are taught throughout the first 2 years. Students advance to the clinical part of the curriculum after passing a high-stakes examination. The clinical phase consists of many individual courses, with the subject of OBGYN being taught in the fifth year. In the first term of that year, students attend lectures on OBGYN, and they complete a 1-week clinical attachment in the following term in groups of 8–11 students each. In the context of the Covid-19 pandemic, the clinical attachment was moved to a virtual space (ILIAS open source e-Learning e. V, Cologne, Germany). The inclusion criteria of the participants were as follows:

- Enrolled medical student at the University of Bonn in the one-week clinical attachment of OBGYN
- Consent to study participation
- Age of the participants > 18 years

The exclusion criteria were as follows:

- Lack of informed consent to participate in the study
- Age of the participants < 18 years

### Course design

During the virtual one-week clinical attachment, each student was given access to a total of four clinical case vignettes—each comprising two obstetric and two gynecological cases. The content presented was aligned to the “Learning Opportunities, Objectives and Outcomes Platform” (LOOOP) of the Medical School Association (Medizinischer Fakultaetentag, MFT), a nationwide online resource for the study of human medicine and dentistry (https://looop-share.charite.de). In addition, a total of 40 short answer questions (SAQs) were written based on the four case vignettes, representing key feature elements of these cases (Table 1).

**Table 1:** Key feature case topics and individual key feature elements

Key feature case	Individual main features
Obstetrics I (case 1)	Determination of the gestational age
	Maternity report
	Indication for induction of labour
	Induction of labour with medication
	Initial examination and admission to the delivery room for delivery
	Evaluation of the results of the vaginal palpation
	Indication for micro blood examination
	Determination of the dilution of a microblood test
	Evaluation of Apgar score and umbilical cord blood gas analysis
	Assessment of the CTG according to FIGO
Obstetrics II (case 2)	Initial examination on admission to the delivery room
	Differential diagnoses for bleeding in the third trimester
	Bishop points
	Assessment of vaginal palpation
	Biometry of the fetus
	Assessment of the CTG according to FIGO
	First measures in case of CTG decelerations
	Measures in case of repeated CTG delays
	Definition of the criteria for the Apgar score
	Evaluation of the Apgar score
Breast carcinoma (case 3)	Imaging procedure for the diagnosis of a breast cancer
	Differential diagnoses of benign breast tumours
	Hereditary risk factors for breast cancer
	Clinical signs of breast cancer
	Risk lesions/pre-cancerous lesions for breast cancer
	Prerequisites for a curative therapeutic approach
	Postoperative approach in an R0 situation
	Metastases in breast cancer
	Important prognostic receptors in breast cancer
	Other carcinomas caused by the BRCA mutation
Cervical carcinoma (case 4)	Definition of heavy menstruation
	Definition of irregular menstruation
	Differential diagnoses for atypical menstrual bleeding
	Primary prevention of cervical carcinoma
	Evaluation of the acetic acid test
	Most frequent histological form of cervical carcinoma
	Metastasis of cervical carcinoma
	Prerequisites for the R0 situation
	Therapy of cervical carcinoma
	Follow-up care for cervical carcinoma

Each student block placement group was exposed to two cases containing key feature questions and two RO cases (i.e., identical content but without KFQs). A reading case consisted of the main text and some background information. A description of the patient's symptoms and history was followed by physical examination findings and results of diagnostic tests as well as information on how the clinical case progressed, including complications. The background information covered aspects that were the subject of KFQs in the corresponding presentation format of the same case.

**Fig. 1:**
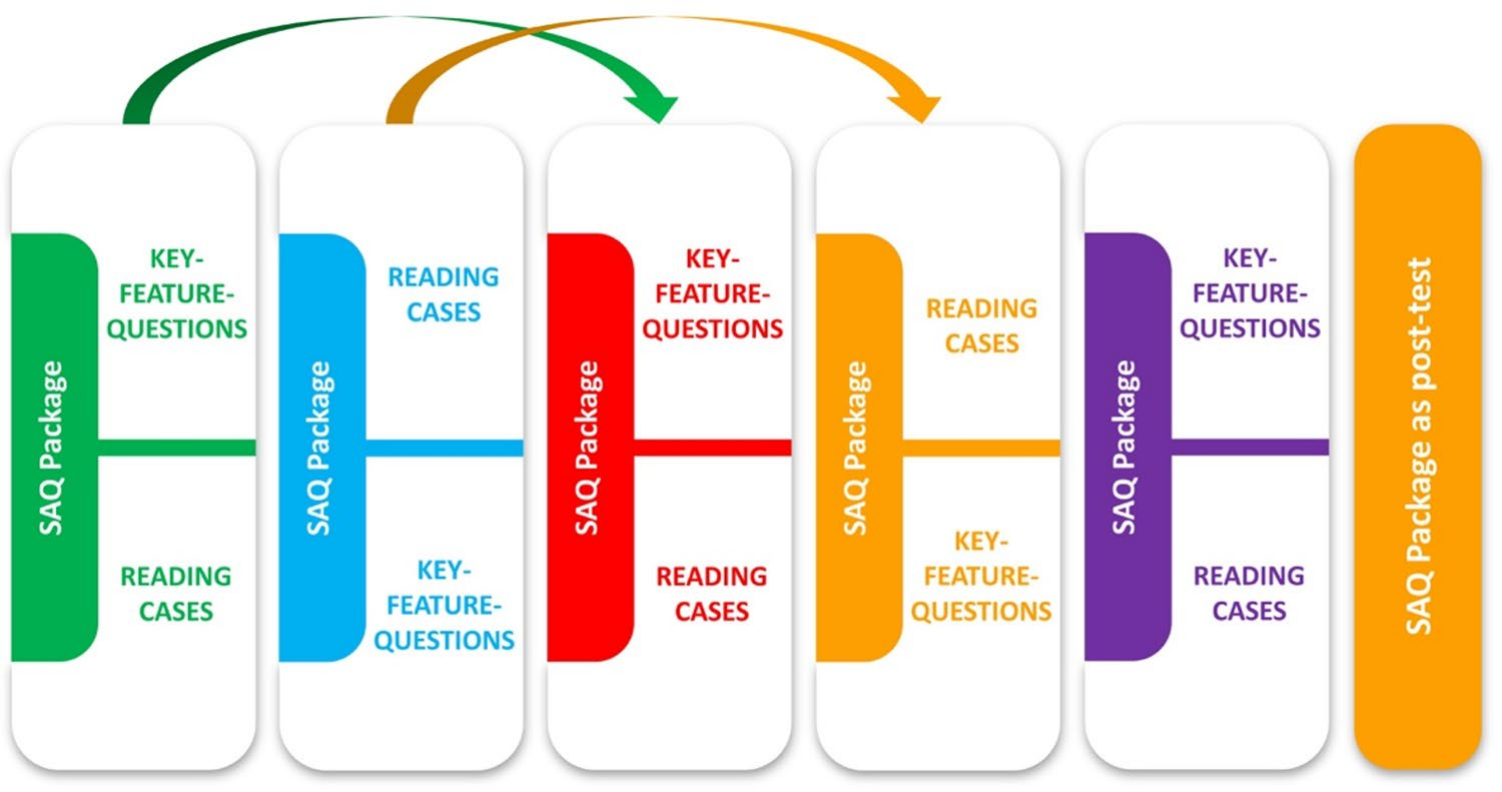
Cross-over study design and case vignettes in the weekly clinical block placement

A specific case that was presented as a RO case to the student group in week 1 was presented as a KF case to the student group in week 2, and vice versa. This was continued in the following weeks, resulting in the cross-over design of the study. As spacing is essential for test-enhanced learning, each group had to revisit their specific key feature cases and reading cases two weeks after their actual virtual clinical attachment. During this process, the students' activities were tracked and recorded in the learning management system. Likewise, each clinical attachment group had to complete the forty SAQ questions as a pre-test at the beginning of their one-week assignment. A total of 60 min was available to complete the test. At the end of term, all students received the same SAQ test as a formative final exam for the subject of gynecology (Fig. 1).

All students participating in the one-week OBGYN clinical attachment in winter term 2020/2021 were required to work on the online cases. On the first day of term, they were invited to provide written consent to participate in the study, i.e. to have their data anonymized and analyzed for study purposes.

### Statistical analysis

Pre- and post-test consisted of 40 SAQ questions each. One specific student would have been exposed to the content covered in 20 of these items in KF cases (‘intervention items’) while the content covered in the remaining 20 items would have been presented in RO cases (‘control items’). For a different student, this assignment would have been the opposite. Dummy coding was used to define intervention and control items for each student, regardless of the group they had been assigned to during the clinical attachment. Descriptive analyses were carried out for the demographic data of the sample as well as the pre-test and post-test. For nominal scaled data, such as demographic variables, simple frequency calculations were carried out for the different characteristics.

A descriptive item or scale analysis was conducted for the 40 items in both the pre-test and the post-test. Cronbach's α was used as a measure of internal consistency.

In order to answer the research question, the differences between percent scores in intervention and control items in the post-test were analyzed using a paired *T*-test.

The second research question was addressed by repeating this analysis for three student groups created particularly for this purpose, each with a different time interval between the intervention and the post-test: Group 1 (G1) had a 1* to 4-week gap from the post-test (*n* = 35), Group 2 (G2) had a 5- to 8-week gap (*n* = 29), and Group 3 (G3) had the longest distance (9–12 weeks) (*n* = 30).

For statistical analysis, IBM SPSS Statistics for Windows version 27.0 was used (IBM Corp., Armonk, NY, USA). Data are presented as mean, standard deviation (SD), or numbers and percentages unless otherwise stated. The alpha level was set at 0.05. This study was approved by the University of Bonn's local ethics commission (application no. 0l4/ 2 l).

## Results

**Fig. 2:**
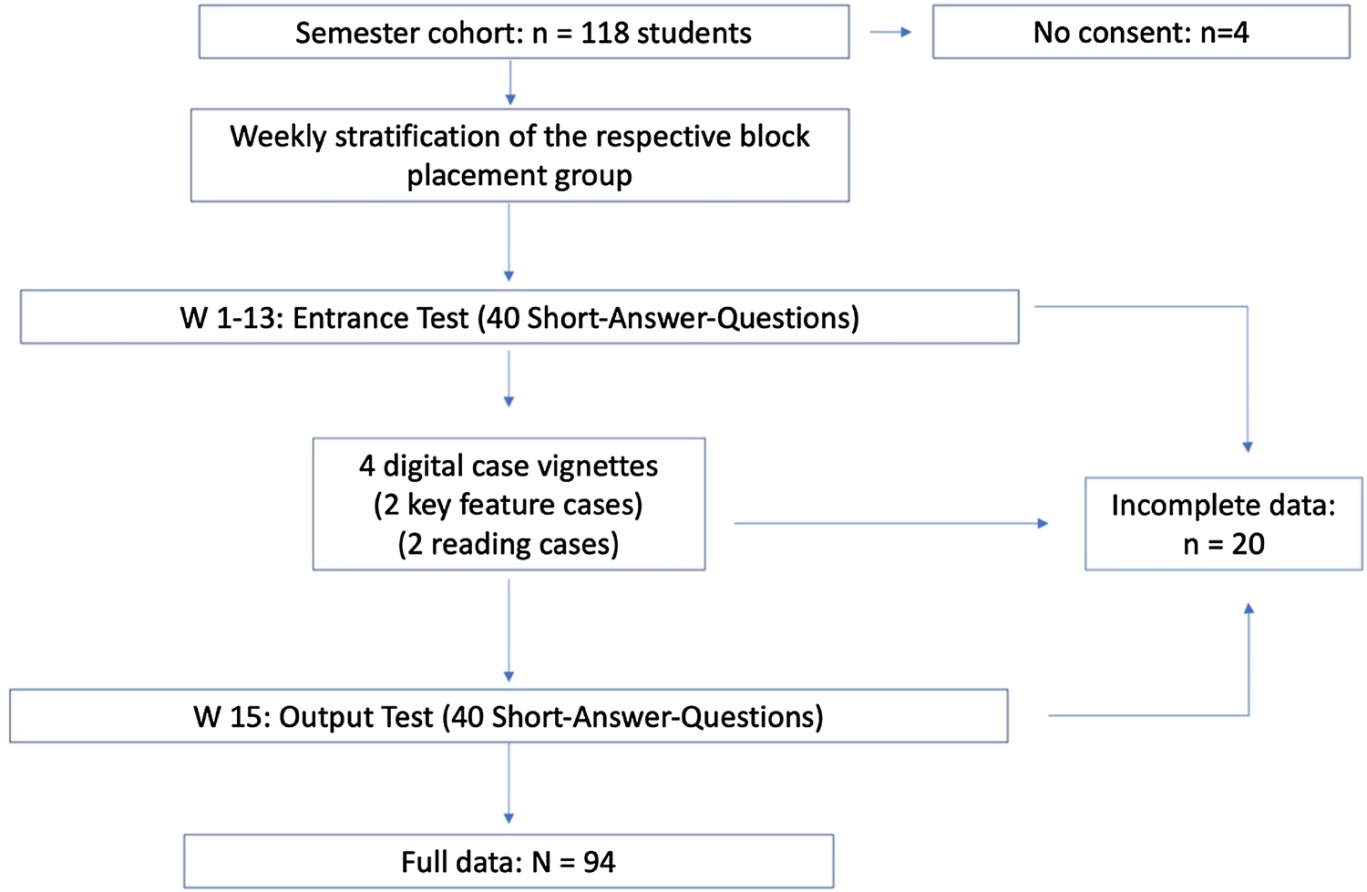
Study cohort and data enrollment

The flow of participants through the study is displayed in Fig. 2. Four of the 118 students who were eligible to participate in the study did not provide written consent. Complete data for 94 students were acquired, yielding an effective response rate of 79.7 percent for this longitudinal sample. 56.4% of the study sample were females. There were no statistically significant differences between the study groups in terms of gender; since the students' dates of birth are not recorded on a personalised basis, a differentiation by age cannot be made.

In order to determine the quality criteria of the pre-test and post-test and thus to be able to assess the measurement quality, the item characteristics were determined for each of the 40 items in both the pre-test and the post-test. These item characteristics for the 40 items in the pre-test and post-test are given in Table 2.

**Table 2:** Item characteristics of the SAQs in the pre- and post-test

Item	Available points	Pre-test			Post-test		
		Mean value ± SD	Discriminatory power	Item difficulty	Mean value ± SD	Discriminatory power	Item difficulty
Determination of the gestational age	2	1.35 ± 0.56	0.273	0.67	1.36 ± 0.50	0.105	0.68
Maternity report	5	2.78 ± 1.34	0.049	0.56	3.80 ± 0.95	0.198	0.76
Indication for induction of labour	1	0.91 ± 0.29	0.165	0.91	0.94 ± 0.14	0.089	0.98
Induction of labour with medication	3	0.37 ± 0.58	0.094	0.12	0.61 ± 0.75	0.142	0.20
Initial examination/admission delivery room	3	0.34 ± 0.52	0.011	0.11	0.40 ± 0.62	0.357	0.13
Evaluation vaginal results vaginal palpation	2	0.74 ± 0.67	0.334	0.37	1.14 ± 0.62	0.241	0.57
Indication for micro blood examination	2	1.91 ± 0.39	0.308	0.95	2.00 ± 0.01	n/a	1.00
Examination of micro blood examination	1	0.20 ± 0.40	−0.027	0.20	0.81 ± 0.40	0.270	0.81
Evaluation of APGAR-score and umbilical cord BGA	2	1.61 ± 0.78	0.162	0.81	1.73 ± 0.59	0.308	0.86
Assessment of the CTG according to FIGO	4	3.31 ± 1.37	0.263	0.83	3.60 ± 0.95	−0.116	0.90
Definition of heavy menstruation	1	0.53 ± 0.50	0.154	0.53	0.51 ± 0.50	0.111	0.51
Definition of irregular menstruation	1	0.75 ± 0.44	0.262	0.75	0.71 ± 0.46	0.105	0.71
Differential diagnoses of atypical bleeding	2	1.41 ± 0.76	0.226	0.71	1.30 ± 0.80	0.230	0.65
Primary prevention of cervical carcinoma	2	1.49 ± 0.50	0.056	0.75	1.39 ± 0.51	0.079	0.70
Evaluation of the acetic acid test	2	1.23 ± 0.72	0.126	0.62	1.24 ± 0.48	0.176	0.62
Most frequent histological form cervical carcinoma*	1	1.00 ± 0.01	n/a	1.00	0.99 ± 0.10	−0.035	0.99
Metastasis of cervical carcinoma	1	0.85 ± 0.36	0.291	0.85	0.93 ± 0.26	0.300	0.93
Prerequisites for the R0 situation	2	1.80 ± 0.43	0.203	0.90	1.72 ± 0.50	0.054	0.86
Therapy of cervical carcinoma	1	0.94 ± 0.23	0.271	0.94	0.98 ± 0.14	0.397	0.98
Follow-up care for cervical carcinoma	2	1.07 ± 0.82	−0.030	0.54	0.91 ± 0.81	0.076	0.45
Initial examination on admission to the delivery room	4	1.63 ± 0.85	0.101	0.41	1.99 ± 0.98	0.239	0.50
Differential diagnoses third trimester bleeding	4	2.21 ± 0.73	0.136	0.55	2.44 ± 0.72	0.229	0.61
Bishop score	1	0.69 ± 0.46	0.090	0.69	0.41 ± 0.50	0.123	0.41
Assessment of vaginal palpation	1	0.77 ± 0.42	0.289	0.77	0.84 ± 0.37	0.352	0.84
Biometry of the fetus	3	0.48 ± 0.87	0.142	0.16	0.10 ± 0.50	0.090	0.03
Assessment of the CTG according to FIGO	1	0.99 ± 0.10	0.143	0.99	0.98 ± 0.14	0.006	0.98
First measures in case of CTG decelerations	1	0.49 ± 0.50	0.058	0.49	0.40 ± 0.49	0.016	0.40
Measures in case of repeated CTG delays	1	0.84 ± 0.37	0.301	0.84	0.65 ± 0.48	0.163	0.65
Definition criteria for the APGAR score	5	4.93 ± 0.53	0.493	0.99	5.00 ± 0.01	n/a	1.00
Evaluation of the Apgar score	1	0.82 ± 0.39	0.292	0.82	0.87 ± 0.34	0.350	0.87
Imaging procedure Breast cancer diagnosis*	1	1.00 ± 0.01	n/a	1.00	1.00 ± 0.01	n/a	1.00
Differential diagnoses benign breast tumours	4	3.61 ± 0.91	0.059	0.90	3.76 ± 0.50	0.229	0.94
Hereditary risk factors for breast cancer	2	1.62 ± 0.69	0.281	0.81	1.87 ± 0.42	0.215	0.93
Clinical signs of breast cancer	3	2.26 ± 0.88	0.173	0.75	2.26 ± 0.86	0.181	0.75
Risk lesions/pre-cancerous lesions Breast cancer	3	2.87 ± 0.39	0.098	0.96	2.88 ± 0.46	0.380	0.96
Prerequisites for curative therapy approach	1	0.84 ± 0.37	0.246	0.84	0.88 ± 0.33	0.101	0.88
Postoperative approach in an R0 situation	1	0.79 ± 0.41	0.335	0.79	0.93 ± 0.26	0.268	0.93
Metastases in breast cancer	3	2.87 ± 0.49	0.179	0.96	2.90 ± 0.42	0.269	0.97
Prognostic receptor factors in breast cancer	3	2.97 ± 0.23	0.217	0.99	2.95 ± 0.26	0.366	0.98
Other carcinomas that are caused by BRCA mutation	1	0.99 ± 0.10	0.056	0.99	0.99 ± 0.10	0.004	0.99

### Primary aim

To answer the primary research question (“Does repeated exposure to clinical cases in OBGYN with built-in key feature questions lead to higher learning success than repeated exposure to cases with the same content but without built-in questions?”), the “intervention” and “control” conditions were analysed independently of the content focus of the items.

**Fig. 3:**
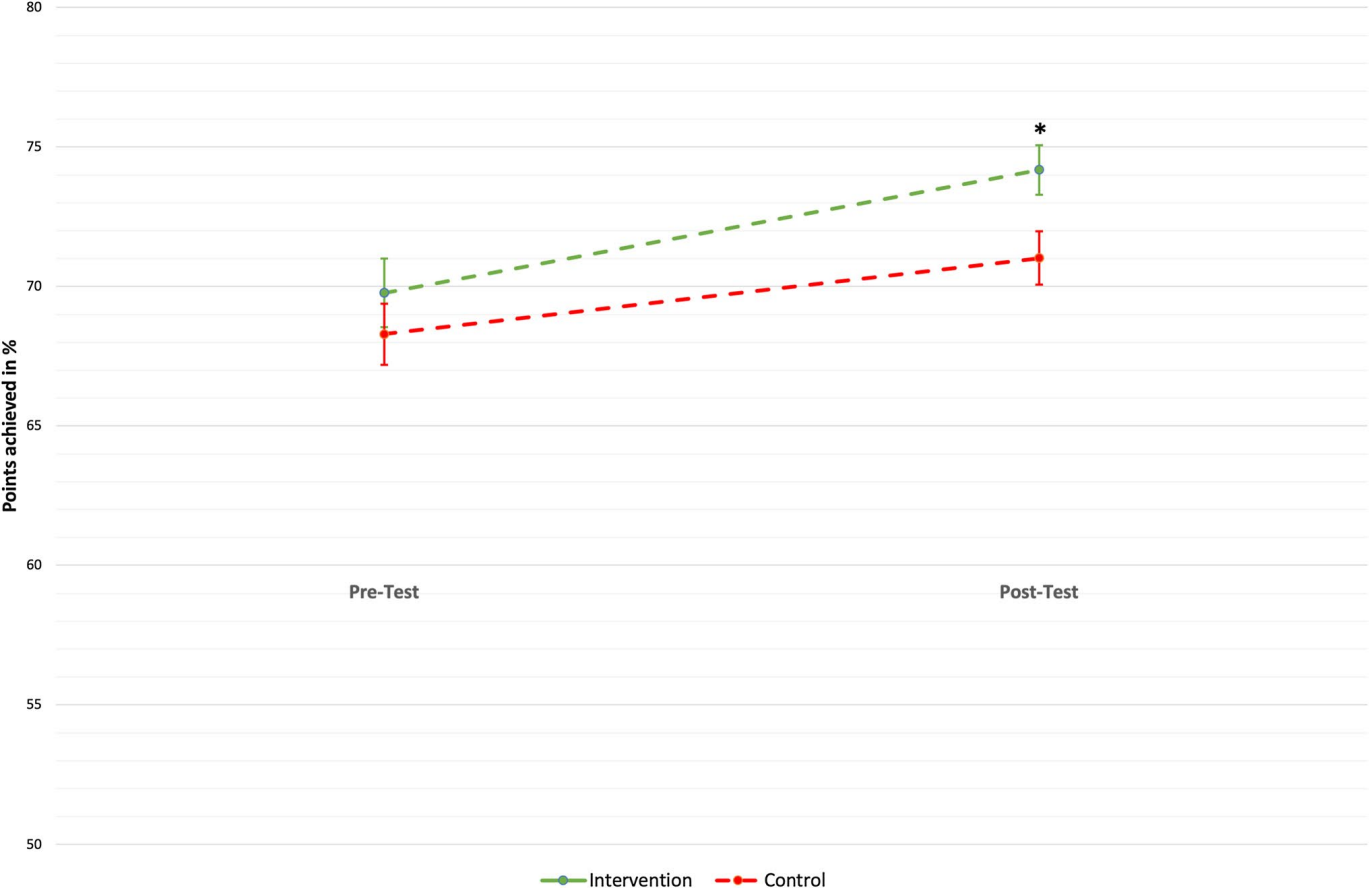
Average scores achieved in % in pre-test and post-test. The error bars represent the standard errors. *p = 0.017 in the paired *t*-test to examine the differences between intervention and control items

In the total sample, an average of 69.3 ± 7.3% of the maximum possible 84 points were achieved in the pre-test and 72.8 ± 6.4% in the post-test. Across groups, 69.8 ± 12.0% of the maximum possible points were achieved in the intervention items in the pre-test and 68.3 ± 10.6% of the maximum possible points in the control items (*p* = 0.410). In the post-test, a mean of 74.2 ± 8.6% of the maximum possible points were achieved for the intervention items and 71.0 ± 9.2% of the maximum possible points for the control items. In the post-test, an average of 3.2 ± 12.4% higher scores in relation to the maximum possible score were achieved in the intervention items than in the control items. This difference in favour of the intervention items compared to the control items proves to be statistically significant (*p* = 0.017) and thus leads to a rejection of the null hypothesis of the primary hypothesis (Fig. 3).

### Secondary aim

To answer the secondary research question two, three groups were formed to determine the influence of the temporal distance between the intervention and the post-test on the outcome: Group 1 (G1) showed a gap of 1–4 weeks to the post-test (*n* = 35), while Group 2 (G2) showed a gap of 5–8 weeks (*n* = 29) and Group 3 (G3) showed the largest gap of 9–12 weeks (*n* = 30). Table 3 displays the results of the analysis for the secondary study question. Following a Bonferroni correction for multiple testing, there was no significant difference in any of the subgroups. However, Table 3 shows very clearly that the retention only decreases significantly for the control items. On average, the intervention items, therefore, not only tend to lead to a higher immediate learning gain, as the higher value for group one shows, but also to a longer persistence of this effect.

**Table 3:** Percent scores in intervention and control items in the post-test by time intervals between the intervention and the post-test

	Intervention items	Control items	*p*
Group 1	74.4 ± 9.2	72.3 ± 10.2	0.274
Group 2	74.0 ± 9.8	71.4 ± 8.7	0.358
Group 3	74.0 ± 6.8	69.3 ± 8.4	0.029

In relation to the secondary research question, there are thus definitely indications that the temporal distance for the control items shows a negative influence on retention. The statistical results, however, are not clear enough to be able to justify the rejection of the null hypothesis in a methodologically and substantively serious manner of the secondary question.

## Discussion

In the present study, the paradigm of repeated testing with KFQs was implemented for the first time in medical students for the consolidation of differential diagnostic and therapeutic competences in the field of gynecology and obstetrics. Despite rather moderate item characteristics, it was shown that the intervention (KF cases) led to greater learning success than the control condition in OBGYN medical education (R0 cases).

Regarding the secondary research question, there was no effect of the delay between the intervention and the final exam on the difference in performance between intervention and control items.

All of the assessments in this study were formative in nature. Because the students did not experience any penalties associated with the assessments, it is reasonable to believe that the indirect testing impact was minor. On the one hand, this avoided the disruptive issue of prospective point loss (and hence a change in learning behavior); on the other hand, this may have had a detrimental influence on the students' diligence in working through the e-case seminars and the final formative assessments.

Repeated testing with key feature questions can be an attractive alternative to more resource-intensive teaching methods for specific learning objectives. Given the scalability associated with e-learning interventions, as well as the pedagogical rationale for using key questions to promote complex cognitive functions, our study contributes to the growing body of literature on how e-learning can be used effectively to improve student learning outcomes [8], especially in pandemics such as the Covid 19 pandemic [8].

Another study examined the use of script concordance examinations in the context of clerkships and the assessment of clinical reasoning in OBGYN [10]. The authors demonstrated satisfactory reliability for assessment in training and a favorable association with the assessment of clinical reasoning using key features.

In line with our null result regarding the impact of the time interval between the intervention and the post-test, a meta-analysis of self-directed learning found no significant relationship between the observed effect size and the duration of the intervention with self-directed learning or the time gap between the conclusion of the intervention and the evaluation of outcomes [11].

A study in the field of gynecological endocrinology showed that case-based learning can be beneficial in postgraduate training [12]. The residents interviewed agreed that case-based, interactive training was superior to traditional lecture-based training. The authors concluded that a non-traditional curriculum can be successfully implemented in a residency training curriculum and significantly improves understanding and confidence in critical endocrinology concepts.

## Limitations

The quality of the SAQ examination items was at best moderate. The data suggest that a number of items was too easy. This could have been avoided by pilot-testing the exam and making necessary adjustments before using the exam in the context of a study [13, 14]. There was no subject-specific focus for the questions that did poorly.

Students' initial level of knowledge was quite high, with a mean score of 69.3%. This might be explained by the fact that the SAQs were focused on knowledge that had already been acquired in the preceding term. According to a literature review, while SAQs have a high discriminatory power due to the possibility of point differentiation and produce tests with high reliability in the digital domain, SAQ correction and evaluation are time-consuming because automatic evaluation of open questions is not possible. Furthermore, to improve clinical reasoning, the study postulates the use of key feature questions rather than SAQ questions as the item to be chosen [14]. In contrast, other studies here point to the use of new methods in the SAQ format, such as the Short Answer Management Problems (SAMPs) format. They are designed to measure a candidate's problem-solving skills and knowledge in the context of a clinical situation and thus strengthen clinical reasoning [15]. New subject-specific assessment rubrics are also being developed for SAQs to strengthen clinical reasoning, such as in the area of manual physical therapy [16].

Despite the statistically significant increase from pre- to post-test scores, there remains doubt that this reflects a clinically meaningful gain in clinical reasoning.

The study's monocentric nature restricts the generalizability of our findings. Because the goal of this study was to give insight into the real-world effectiveness of test-enhanced learning, certain potential confounding factors were not experimentally controlled. Most crucially, we did not gather data on how much time was spent on self-study. Unlike laboratory studies of test-enhanced learning, we did not try to limit the amount of time students spent on case vignettes, but instead allowed them to complete their sessions whenever they wanted. Finally, this study did not investigate whether repeated testing with KFQs impacted on students' clinical performance. Although one study suggests an association, further research is needed to establish a causal link between frequent testing and improved patient outcomes [17].

### Conclusion

Our data demonstrate improved retention following repeated formative testing with KFQs in obstetrics and gynecology. The time interval between the intervention and the point of final data collection did not mediate this effect.

## Data Availability

Not applicable.
